# Notes and News

**Published:** 1897-09-11

**Authors:** 


### Notes and News.

(Collected and Communicated.)

Another use for the bicycle has now been found. According to one of our contemporaries an ambulance bicycle is being built in London. It consists of two bicycles braced together at such a distance that a stretcher on springs can swing between them. The bicycles differ only slightly from the ordinary machine. Each half of the machine is to be provided with a holder for the ambulance requisites, and it is stated that the bicycle ambulance will cost rather less than the one now in use.

The cottage hospital is spreading in popularity, and Mr. Brunker, Acting Premier of New South Wales, when opening a cottage hospital in July, on Botany Bay, remarked that a small, manageable institution, built on lines at once pretty and convenient, would meet with more success in the future than the larger hospitals. The St. George's Cottage Hospital, like larger institutions for the sick in New South Wales, is to be assisted by Government. The first part of the building was opened in 1894. It stands on a beautiful site three and a half acres in extent.

What is known as the "Badge" system is this year to be adopted in connection with the Hospital Saturday movement in aid of the Essex and Colchester Hospital. Badges are to be on sale on Hospital Saturday as well as in shops on days previously, which can be purchased for not less than 1s. each, and as much more as purchasers care to give. These should be worn by all the owners on Hospital Saturday, and the wearing of such a badge will ensure that the wearers will not be asked to contribute to any of the boxes. It is expected that the sale of these badges will realise nearly twice as much as was collected in Colchester on Hospital Saturday last year. It remains to be seen whether the inhabitants of Colchester consider their freedom from the importunities of street collectors cheaply bought at 1s., or otherwise. The authorities of the Colchester Hospital Saturday organisation frankly state their intention of "bothering" every person not wearing such a badge.

The treasurer of Guy's Hospital has received a donation of £1,000 from the Committee of the American Victoria Jubilee Fund to endow in perpetuity a "Queen Victoria Bed." A like amount for the same object has also been given by the American Victoria Jubilee Fund to Charing Cross and the London Hospitals.

The Bristol General Hospital has received a gift from a generous friend of £1,500 towards defraying the cost of the enlargement and reconstruction of the laundry. Although it will cost much more than the £1,500 given, the work is to be put in hand with all speed, and the committee appeal for more funds to enable them to complete these necessary alterations and enlargement.

The Bengal College of Physicians and Surgeons was formally opened on July 22nd. The institution is not dependent on a Government grant, but has been organised and carried on by a few private practitioners, its chief object being to further medical knowledge by the scientific training of students. The college is well fitted with all the newest apparatus for the study of every branch of medicine and surgery, and there is an exhaustive library.

The Honorary Secretaries of the Prince of Wales's Hospital Fund for London have received from Mr. W. Y. Campbell, of Johannesburg, S.A.R., the generous contribution of £1,602 5s. 3d., being collections as follows: Witwatersrand Celebration of the Queen's Record Reign by British Residents of Witwatersrand, Transvaal, per Mr. W. Y. Campbell, £1,452 5s. 3d.; Britons of Potchefstroom, per Mr. Mountfield, £120; Nigel Gold Mining Company (Limited), per Mr. A. Grant, general manager, and employés, £30.

The governors of the Newcastle Royal Infirmary have received a munificent offer from Mr. John Hall. If, instead of rebuilding upon the present site, which is in the centre of the town, they will decide upon the erection of a new infirmary upon the Leazes, or upon or near to the Recreation Ground on the North Road, he will defray the cost of a new building, and of fitting and furnishing it, within the limit ot £100,000, on condition that the subscriptions to the New Infirmary Building Fund, which already amount to about £100,000 shall become available for the general purposes of the institution.

Ambulance classes for sailors are now much encouraged. The fact that the Merchant Shipping Act does not require vessels carrying under one hundred persons to carry a medical officer, renders it very necessary that some amongst the crew of smaller craft should know some of the simpler methods of treating accidents. The Trinity House offers silver badges to all their men who pass the test of the Ambulance Association, and classes are organised by the Shipmasters' Association and other institutions. A very large number of seamen avail themselves of the opportunities offered them to pass the examinations, and it is to the true interest of all shipowners to afford facilities to the men they employ to undergo such useful instruction.

